# Genetic redundancy in the naphthalene-degradation pathway of *Cycloclasticus pugetii* strain PS-1 enables response to varying substrate concentrations

**DOI:** 10.1093/femsec/fiae060

**Published:** 2024-04-13

**Authors:** Anjela L Vogel, Katharine J Thompson, Daniel Straub, Florin Musat, Tony Gutierrez, Sara Kleindienst

**Affiliations:** Eberhard Karls University of Tübingen, Department of Geosciences, Schnarrenbergstr. 94-96, Tübingen 72076, Germany; University of Stuttgart, Department of Environmental Microbiology, Institute for Sanitary Engineering, Water Quality and Solid Waste Management (ISWA), Am Bandtäle 2, Stuttgart 70569, Germany; Eberhard Karls University of Tübingen, Department of Geosciences, Schnarrenbergstr. 94-96, Tübingen 72076, Germany; University of Stuttgart, Department of Environmental Microbiology, Institute for Sanitary Engineering, Water Quality and Solid Waste Management (ISWA), Am Bandtäle 2, Stuttgart 70569, Germany; Eberhard Karls University of Tübingen, Quantitative Biology Center (QBiC), Auf der Morgenstelle 10, Tübingen 72076, Germany; Cluster of Excellence: EXC 2124: Controlling Microbes to Fight Infection, Auf der Morgenstelle 28, Tübingen 72076, Germany; Aarhus University, Department of Biology, Section for Microbiology, Ny Munkegade 116, Aarhus C 8000, Denmark; Babeş-Bolyai University, Department of Molecular Biology and Biotechnology, Faculty of Biology and Geology, Str. Republicii nr 44, Cluj-Napoca 400015, Romania; Heriot-Watt University, Institute of Mechanical Process and Energy Engineering (IMPEE), School of Engineering and Physical Sciences, Edinburgh EH14 4AS, UK; Eberhard Karls University of Tübingen, Department of Geosciences, Schnarrenbergstr. 94-96, Tübingen 72076, Germany; University of Stuttgart, Department of Environmental Microbiology, Institute for Sanitary Engineering, Water Quality and Solid Waste Management (ISWA), Am Bandtäle 2, Stuttgart 70569, Germany

**Keywords:** effect of PAH concentration, marine biodegradation, ring-hydroxylating dioxygenases, substrate-independent expression of PAH-degradation genes

## Abstract

Polycyclic aromatic hydrocarbon (PAH) contamination in marine environments range from low-diffusive inputs to high loads. The influence of PAH concentration on the expression of functional genes [e.g. those encoding ring-hydroxylating dioxygenases (RHDs)] has been overlooked in PAH biodegradation studies. However, understanding marker-gene expression under different PAH loads can help to monitor and predict bioremediation efficiency. Here, we followed the expression (via RNA sequencing) of *Cycloclasticus pugetii* strain PS-1 in cell suspension experiments under different naphthalene (100 and 30 mg L^−1^) concentrations. We identified genes encoding previously uncharacterized RHD subunits, termed *rhdPS1α* and *rhdPS1β*, that were highly transcribed in response to naphthalene-degradation activity. Additionally, we identified six RHD subunit-encoding genes that responded to naphthalene exposure. By contrast, four RHD subunit genes were PAH-independently expressed and three other RHD subunit genes responded to naphthalene starvation. *Cycloclasticus* spp. could, therefore, use genetic redundancy in key PAH-degradation genes to react to varying PAH loads. This genetic redundancy may restrict the monitoring of environmental hydrocarbon-degradation activity using single-gene expression. For *Cycloclasticus pugetii* strain PS-1, however, the newly identified *rhdPS1α* and *rhdPS1β* genes might be potential target genes to monitor its environmental naphthalene-degradation activity.

## Teaser

This paper investigates how PAH-degrading bacteria may respond to varying levels of contamination through gene expression analysis for the first and most relevant step in naphthalene degradation.

## Introduction

Polycyclic aromatic hydrocarbons (PAHs) are classified as substances of concern (Environmental Protection Agency 1993) and are ubiquitous in marine environments where they bio-accumulate and are toxic to sea life as well as humans (Landrum et al. 2003, Nikolaou et al. 2009, Murawski et al. 2014, González-Gaya et al. 2016, Stading et al. 2021, Zhang et al. 2021). The biodegradation of these chemicals, principally by microorganisms, is a crucial process in their oxidation, which ultimately mitigates their toxicity effects (National Research Council 2003, Genovese et al. 2014, Duran and Cravo-Laureau 2016, Overholt et al. 2016, González-Gaya et al. 2019). Microorganisms are involved in PAH biodegradation, either by performing one step of the degradation pathway as part of a community or by using the complete pathway for the full degradation of one or more of these types of chemicals (Dombrowski et al. 2016, Joye et al. 2016, Gutierrez 2019a, Mahjoubi et al. 2021). *Cycloclasticus* spp. are known key PAH degraders commonly found in contaminated marine habitats and can completely oxidize PAHs like naphthalene, phenanthrene and pyrene (Kasai et al. 2002, Cui et al. 2014, Wang et al. 2018, Bagi et al. 2022). Naphthalene, as the PAH with the highest water solubility, is often used as a model compound in PAH biodegradation studies, and its biodegradation pathway in *Cycloclasticus* spp. has been well described (Fig. S1) (Wang et al. 2018, 2021).

To date, multiple genes involved in naphthalene degradation have been discovered and more candidate genes likely exist in the genome of the model organism *Cycloclasticus pugetii* strain PS-1. However, it is important to consider that there are still knowledge gaps in our understanding of the naphthalene- and, more generally, PAH-degradation pathways occurring in marine habitats. For example, Sieradzki et al. (2021) could not detect a complete naphthalene-degradation pathway in whole community metagenomes from PAH-contaminated surface water samples. Additionally, there is a paucity of insight into the transcriptional behavior and the factors influencing the expression of functional genes. A recent paper using metatranscriptomics revealed species-specific responses of two key hydrocarbon degraders, *Marinobacter* and *Colwellia*, to distinct exposure regimes resulting from additions of organic carbon derived from oil, synthetic dispersant, or oil and synthetic dispersant (Pena-Montenegro et al. 2023). In the environment, PAH emissions from anthropogenic sources range from low diffusive inputs (e.g. through transportation and river runoff) to high amounts (e.g. through shipping, oil pipelines and platform/rig accidents) (National Research Council 2003, Duran and Cravo-Laureau 2016, Ryther et al. 2021). Considering the wide range of emitted PAH loads, one factor overlooked so far in PAH biodegradation studies is the influence of environmental PAH concentrations in inducing gene expression for each of the biodegradation pathway steps.

One important application of investigating the PAH-degradation pathway is to predict and monitor a microbial ecosystem’s response to PAH contamination. This response is dependent on the set of functional genes in the microbial community and the conditions that result in their expression. The genes encoding key enzymes in the PAH-degradation pathway could impact degradation differently, depending on their transcriptional behavior. Given the potential correlation between transcription and PAH-degradation activity, functional marker genes, which are transcribed dependent on available substrate, could help to develop a quantitative PCR (qPCR)-based tool (i.e. assays targeting the transcript-to-gene ratio) serving as a measure for environmental PAH-degradation activity (Wilson et al. 1999, Baelum et al. 2008, Brow et al. 2013, Tentori and Richardson 2020, Vogel et al. 2023b ). However, it is unknown if there is naphthalene-concentration-dependent marker-gene expression for functional genes in *Cycloclasticus pugetii* strain PS-1.

Genes that are well conserved in PAH-degrading organisms (Meynet et al. 2015, Liang et al. 2019) can be used as marker genes to identify key PAH degraders in environmental communities through DNA-based analyses (Genovese et al. 2014, Bagi et al. 2022), even if they are transcribed independently of PAH availability. Because the expression of such substrate-independently-expressed genes does not reflect the PAH-degradation activity, transcriptomic results must be interpreted with caution. In a previous study, we found that *Cycloclasticus pugetii* strain PS-1 expressed three functional genes involved in PAH degradation independent of substrate availability (Vogel et al. 2023b); however, the transcriptional pattern of other genes of *Cycloclasticus pugetii* strain PS-1 is lacking. Furthermore, whether substrate-independent expression of functional PAH-degradation genes is a common strategy in *Cycloclasticus pugetii* strain PS-1 remains unconstrained.

Upon further examination of the genomes of *Cycloclasticus* spp., multiple genes encoding enzymes that are potentially capable of performing the same reaction in the naphthalene-degradation pathway exist, hinting at genetic redundancy (Wang et al. 2018, Bagi et al. 2022). Multiple layers of functional redundancy exist in microorganisms (Ghosh and O'Connor 2017), and while functional redundancy seemingly counters selective pressure (Nowak et al. 1997), it could lead to flexibility and thus be a benefit for the organism (Laruson et al. 2020). However, it is unknown if there is genetic redundancy in the naphthalene-degradation pathway, if there is one preferred gene or a set of genes for each step of the naphthalene-degradation pathway, and which conditions select these. In this study, we sought to determine if *Cycloclasticus pugetii* strain PS-1 possesses functional marker genes that are transcribed during naphthalene active degradation, or if substrate-independent expression of PAH-degradation genes is a common strategy in this organism. We also examined whether genetic redundancy for genes involved in the naphthalene-degradation pathway occurs in this organism.

## Materials and methods

### *Cycloclasticus pugetii* strain PS-1 cell suspension experiments

A freeze-dried culture of the well-studied PAH-degrading marine model organism *Cycloclasticus pugetii* strain PS-1 [American Type Culture Collection (ATCC) 51542], originally isolated from deep-sea sediments of the Pacific Ocean in Puget Sound (Dyksterhouse et al. 1995), was acquired from the ATCC (Virginia, USA). Strain PS-1 was revived according to the manufacturer's instructions and maintained in liquid culture with Marine-Bouillon 2216 (Sigma-Aldrich, USA), supplemented with 100 mg L^−1^ naphthalene. The cultures were confirmed as *Cycloclasticus pugetii* strain PS-1 with Sanger sequencing of the 16S rRNA gene, prior to conducting the RNA-sequencing experiment.

The experiments were conducted using a cell suspension in late log phase growth with a high cell density rather than a growing culture to eliminate growth as a parameter and thereby enable comparing the transcriptional response of *Cycloclasticus* with different PAH concentrations. To prepare inocula of cell suspensions, 700 ml of day 4 pre-culture was pooled by centrifugation (5000 xg; 10 min), and then the cell pellet was washed twice with fresh medium prior to resuspending (by vortexing) to 70 ml, resulting in a highly concentrated suspension of strain PS-1 cells. To set up the cell suspension experiment, 800 µL of the highly concentrated *Cycloclasticus pugetii* strain PS-1 inoculum was added to individual 20-ml serum vials containing a total of 8 ml of carbon- and nutrient-rich artificial seawater medium (Difco 2216, Sigma-Aldrich, USA), resulting in high cell densities of the order of 10^8^ cells mL^−1^ (Fig. S2). Naphthalene was provided at two loads, 30 and 100 mg L^−1^, which were selected to represent two concentrations from near and above the solubility of this chemical in seawater [28.96 mg L^−1^ (Vogel et al. 2023a )]. The first concentration, in low-NAP and pulse-NAP treatments, is near naphthalene's solubility in seawater and mimics diffusive low-concentration PAH input into marine environments. The added amount was expected to dissolve in the culture medium, so contaminant consumption would lead to a continuous decrease of naphthalene concentrations up to its depletion. In the pulse-NAP treatments, a second low-concentrated pulse of 30 mg L^−1^ was added at 71 h after complete degradation of the initial naphthalene. In high-NAP conditions, the addition of naphthalene above its water solubility was expected to act as a substrate reservoir. As naphthalene was consumed, more would dissolve from crystals, thus maintaining exposure of cells to relatively constant naphthalene concentrations (similar to a steady state set-up) (Vogel et al. 2023a). The reservoir was depleted once the total naphthalene concentration decreased below the water solubility limit. We considered that this experimental condition mimicked massive oil spills, which will infuse large amounts of hydrocarbons into seawater, often at concentrations above water solubility. Therefore, 53 µL, 16 µL, or two times 16 µL of a highly concentrated naphthalene stock solution (15 227.87 mg L^−1^ dissolved in acetone) were added to final concentrations of 100 mg L^−1^ (high-NAP) and 30 mg L^−1^ (low-NAP), or as two pulses of 30 mg L^−1^ (pulse-NAP), respectively. The acetone evaporated immediately, leaving the naphthalene concentration near its solubility in seawater (28.96 mg L^−1^) and visible as undissolved crystals (Vogel et al. 2023a). The same steps were undertaken to prepare uninoculated vials with naphthalene as abiotic controls. Naphthalene concentrations in the inoculated and uninoculated vials were quantified (described below) over the course of the experiment.

For preparation of a PAH-free control, 200 µL of 0.1 M pyruvate was added to individual 20-ml serum vials that contained 8 ml of medium. This addition was comparable with the molar amount of carbon (0.001 mol L^−1^) added in the high-NAP set-up. All biotic vials were set up as three sacrificial samples per timepoint for naphthalene quantification, and three additional sacrificial bottles for DNA and RNA extraction, which were all incubated in the dark on a rotary shaker (125 r/m; 18°C). Sacrificial bottles were used to avoid mass losses of naphthalene due to volatilization. A liquid-liquid extraction of each bottle, with the strong solvent cyclohexane, was used to fully extract the total naphthalene from both the sorbed phase and the aqueous phase. Naphthalene was quantified after inoculation, as well as after 12, 24, 48 and 168 h for high-concentration treatments, and once in the PAH-free controls after 24 h. PAHs were quantified in low-concentration treatments after inoculation and after 12, 24 and 71 h. Pulsed treatments had identical conditions to low-NAP treatments between 0 and 71 h and, therefore, were only sampled after 73, 85, 97 and 168 h. Samples for RNA-sequencing were taken 2 h after inoculation for all naphthalene-containing treatments, including following the second pulse of naphthalene in the pulse-NAP set-ups, and at a later nutrient-depleted (“starvation”) timepoint when naphthalene was fully degraded.

### Quantification of hydrocarbons

Naphthalene concentrations were quantified by gas chromatography coupled to mass spectrometry (GC-MS). A liquid-liquid extraction with cyclohexane of each complete sacrificial bottle was performed to avoid mass losses of naphthalene due to volatilization. As an internal standard, 12.6 mg L^−1^ of D_8_-naphthalene (dissolved in acetone) was added into the crimped vials before using 10 ml of cyclohexane as an organic solvent to extract naphthalene. Subsequently, the samples were shaken for 35 min at 270 r/m and the organic and aqueous phases were allowed to separate undisturbed for 48 h in the dark. The organic phase was subsequently extracted using a glass syringe, and diluted 1:100 with cyclohexane, before naphthalene was quantified using an Agilent 6890 N GC coupled to an Agilent 7973 inert MS. The GC-MS was equipped with an Agilent 7683 B autosampler with a J + W Scientific DB-5MS capillary column (30-m length; 0.025-mm ID; 0.25-µm film thickness) and was operated in single ion mode with splitless injection and a helium flow rate of 0.8 ml min^−1^.

### DNA and RNA extraction and processing

For quantification of the transcription of PAH-related genes during active naphthalene degradation (2 h after addition of naphthalene) and under starving naphthalene conditions, RNA sequencing was conducted. The timepoints for sampling were chosen based on previous experiments (Vogel et al. 2023b). Hence, for DNA and RNA analyses, the total volume (8 ml) of each sacrificial bottle was filtered through sterile 0.22-µm Sterivex filters (Merck Millipore, Darmstadt, Germany) on ice and stored at -80°C until further processing. DNA and RNA were extracted using the Allprep mRNA/DNA kit (Quiagen, Hilden, Germany) according to the manufacturer's instructions with the exception that the extraction buffer was added directly to the Sterivex cartridges, and these were then vortexed at medium power for 4 min before removing the buffer with a 10-ml syringe and transferring it to the first spin column. Immediately upon completing these extraction steps, DNA was stored at -20°C and mRNA at -80°C until further analysis. For RNA purification, the TURBO DNA-free kit (Thermo Fisher Scientific Inc., USA) was used to digest any remaining DNA, following the manufacturer's instructions. The resulting DNA-free RNA was submitted to the Institute for Medical Microbiology and Hygiene (University of Tübingen) for library preparation using Illumina stranded RNA prep, rRNA depletion with Ribo-zero Plus, and sequencing using NextSeq 500 High Output Kit v2.5 (75 cycles, Illumina, San Diego, CA, USA).

### Monitoring cell numbers—qPCR of functional genes *rhd3α* and *rhd2α*

To ensure the cell density remained constant over the course of the cell suspension experiment, we followed two functional genes—*rhd3α* and *rhd2α*—using qPCR. Both genes encode alpha subunits of aromatic ring-hydroxylating dioxygenases (RHD2α and RHD3α) and are present only once per genome in *Cycloclasticus pugetii* strain PS-1 (Vogel et al. 2023b). Both qPCR methods and primers were already developed and used elsewhere: *rhd3α* by Dionisi et al. (2011) (note that the gene is referred to as *phnA1* in this study) and *rhd2α* in our previous study (Vogel et al. 2023b). Primer and qPCR protocol information can be found in Vogel et al. (2023b) (see also Tables S1 and S2).

### Bioinformatic analysis

To analyze the RNA-sequence data, an index database, adjusted to small genomes (genomeSAindexNbases 4), was created with Spliced Transcripts Alignment to a Reference (STAR) v2.6.1d (Dobin et al. 2013) using the *Cycloclasticus pugetii* PS-1 genome assembly ASM38441v1 from NCBI (RefSeq assembly accession GCF_000384415.1). Based on the created reference genome index, nf-core/rnaseq v1.4.2 (https://nf-co.re/rnaseq) (Ewels et al. 2020), and its containerized software, was used with singularity v3.4.1 (Kurtzer et al. 2017) and executed with Nextflow v21.10.3 (Di Tommaso et al. 2017). Nf-core/rnaseq performed quality checks using FastQC v0.11.8, a quality control tool for high-throughput sequence data that is available online at http://www.bioinformatics.babraham.ac.uk/projects/fastqc/ (Andrews 2010). Additionally, <0.5% of basepairs per sample were removed due to adapter contamination and trimming of low-quality regions with Trim Galore! V0.6.4. Up to 11.4% to 35.4% rRNA sequences were removed with SortMeRNA v2.1b (Kopylova et al. 2012). Using STAR v2.6.1d, 81.38% to 95.62% reads were aligned, and, finally, 6.0 to 12.3 million counts per sample (total: 225 986 447; average: 9 416 101 reads) were assigned to genes by featureCounts v1.6.4 (Liao et al. 2014).

To compare the differences in total gene expression between the different set-ups, multidimensional scaling (MDS) was conducted. MDS based on expression profile distances of the top 500 log2-fold changes between sample pairs with edgeR v3.26.5 was plotted for all treatments (Robinson et al. 2010). To assess the differences in the transcription levels of each gene, gene counts were used in differential abundance analysis for all treatments in R v4.1.1 (2021–08–10) with DESeq2 v1.34.0 (Love et al. 2014) using singularity container https://depot.galaxyproject.org/singularity/bioconductor-deseq2:1.34.0--r41h399db7b_0. Significant differences were postulated for transcripts using the Benjamini and Hochberg-adjusted *P*-value (*P*_adj_) ≤ 0.05. Finally, gene counts were transformed to transcript per million (TPM) to allow for the comparison of gene expression between treatments with StringTie2 v2.1.7 (Kovaka et al. 2019).

To identify key metabolic functions in the genome of strain PS-1, functional hidden Markov model profile-based KEGG orthology (KO) annotation and KEGG mapping (Kanehisa and Goto 2000, Kanehisa et al. 2016a,b) was conducted using KofamKOALA v2022-06-02 (Kanehisa et al. 2016a,b, Aramaki et al. 2020) with a threshold E-value = 0.01, with release 102.0 (https://www.genome.jp/tools/kofamkoala/). All genes were grouped by functional categories using the KEGG database “modules” (level 3) while omitting any unannotated genes, and cumulative mean TPM of the biological triplicates were plotted per functional category.

### Identification of the genes involved in PAH degradation and defining transcription categories

To find candidate genes for all the reactions involved in the naphthalene-degradation pathway, we compiled a database containing 154 *Cycloclasticus pugetii* strain PS-1 genes related to PAH degradation (Table S3, sheet A) using the annotations of the NCBI database (August 2022) (Schoch et al. 2020), the KEGG database (August 2022) (Kanehisa and Goto 2000, Kanehisa et al. 2016a,b) and published literature (Wang et al. 1996, Kasai et al. 2003, Wang et al. 2018, Liang et al. 2019, Wang et al. 2021, Bagi et al. 2022).

A sub-set of PAH-related genes was generated from the curated 154 PAH-gene literature-database (described above; Table S3, sheet A) using R (v3.6.0 (2019–04–26) and Rstudio 2022.07.2+576). Genes of interest were selected based on high expression under naphthalene-containing conditions (mean TPM in at least one of the naphthalene-containing treatments within the 90th percentile, mean TPM ≥ 451). The resulting 43 genes (Table S3, sheet B) were assigned to the categories defined below, sorted by pathway step, and the TPMs, as a measure of expression, were plotted in a heatmap. Furthermore, the significance between the naphthalene-containing conditions and the no-PAH control was highlighted (genes with -1 < log2-fold change < 1 and *P*_adj_ ≤ 0.05).

To identify patterns in gene expression pertaining to naphthalene availability, four categories based on the genes’ transcription in the presence or absence of naphthalene (NAP_pos_, NAP_neg_, NAP_indep_, no pattern) were defined (Table S4), as follows: (i) significant upregulation under naphthalene-containing conditions and/or downregulation under naphthalene-starvation conditions compared with the no-PAH control (NAP_pos_); (ii) significant downregulation in naphthalene-containing conditions and/or upregulation under naphthalene-starvation conditions compared with the no-PAH control (NAP_neg_); (iii) genes that showed no significant upregulation or downregulation in the naphthalene-containing treatments (NAP_indep_) compared with the no-PAH control; and (iv) genes with no clear pattern in upregulation or downregulation, irrespective of the naphthalene concentration (no pattern).

## Results

### Hydrocarbon degradation

**Figure 1.**
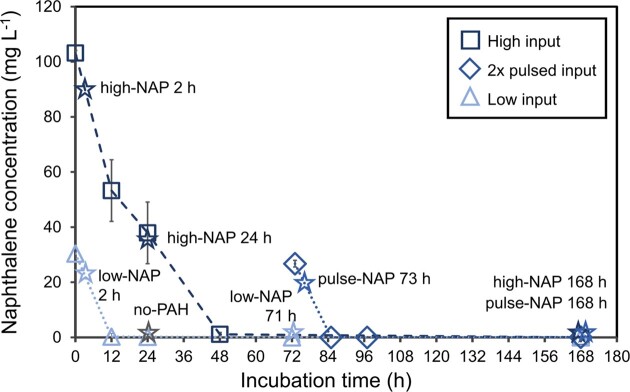
Naphthalene concentration in mg L^−1^ over time, quantified by GC-MS. Squares show concentrations in high-NAP (100 mg L^−1^), lighter blue triangles illustrate concentrations in low-NAP (one dosage; 30 mg L^−1^) and darker blue diamonds give the concentrations in the pulse-NAP treatments (2×30 mg L^−1^). Pulsed treatments were treated equally to low-NAP treatments up until 71 h and, therefore, only sampled after 73, 85, 97 and 168 h. Error bars represent the standard deviation of the respective three sacrificial samples and are sometimes within the marker. Timepoints for RNA-sequencing are marked with stars and colored according to treatments.

Naphthalene was fully degraded, regardless of the starting concentration, by cell suspensions of *Cycloclasticus pugetii* strain PS-1 over 168 h (Fig. 1), while cell numbers remained constant—between 1.28 × 10^8^ and 4.66 × 10^8^ cells L^−1^—over the course of the experiment (Fig. S2). In high-NAP treatments, degradation of 103.2 ± 0.93 mg L^−1^ naphthalene to 1.12 ± 0.70 mg L^−1^ was observed within 48 h. A maximum degradation rate of 4.16 mg L^−1^ h^−1^ was reached within the first 12 h, followed by a decrease in degradation activity between 12 and 48 h (rates for each 12-h interval were 1.28 and 1.53 mg L^−1^ h^−1^, respectively). The residual naphthalene was fully degraded during the remaining incubation time. In both low-NAP (30.4 ± 0.58 mg L^−1^) and pulse-NAP (26.73 ± 0.33 mg L^−1^) treatments, the complete degradation of naphthalene occurred within 12 h of inoculation and after pulsing, with a degradation rate of 2.51 and 2.23 mg L^−1^ h, respectively. Naphthalene concentrations in all abiotic controls remained constant over the course of the experiment and no naphthalene was detected in PAH-free controls (Table S5).

### Overall transcriptional activity in *Cycloclasticus pugetii* strain PS-1

To study gene expression profiles, we assessed similarities in log2-fold changes between samples. MDS between gene expression profiles showed that the transcription intensity within treatments (conducted in biological triplicates) was much more similar than between treatments (Fig. S3). Naphthalene concentration in the samples and the time elapsed following the addition of PAH could explain the difference in transcription between the samples. Correspondingly, transcription in all samples where naphthalene had been completely consumed (starvation) was similar, regardless of the initial substrate concentration. The 2-h low-NAP and pulse-NAP treatment transcriptomes also grouped together and the high-NAP samples after 2 h grouped separately from them (Fig. S3).

**Figure 2.**
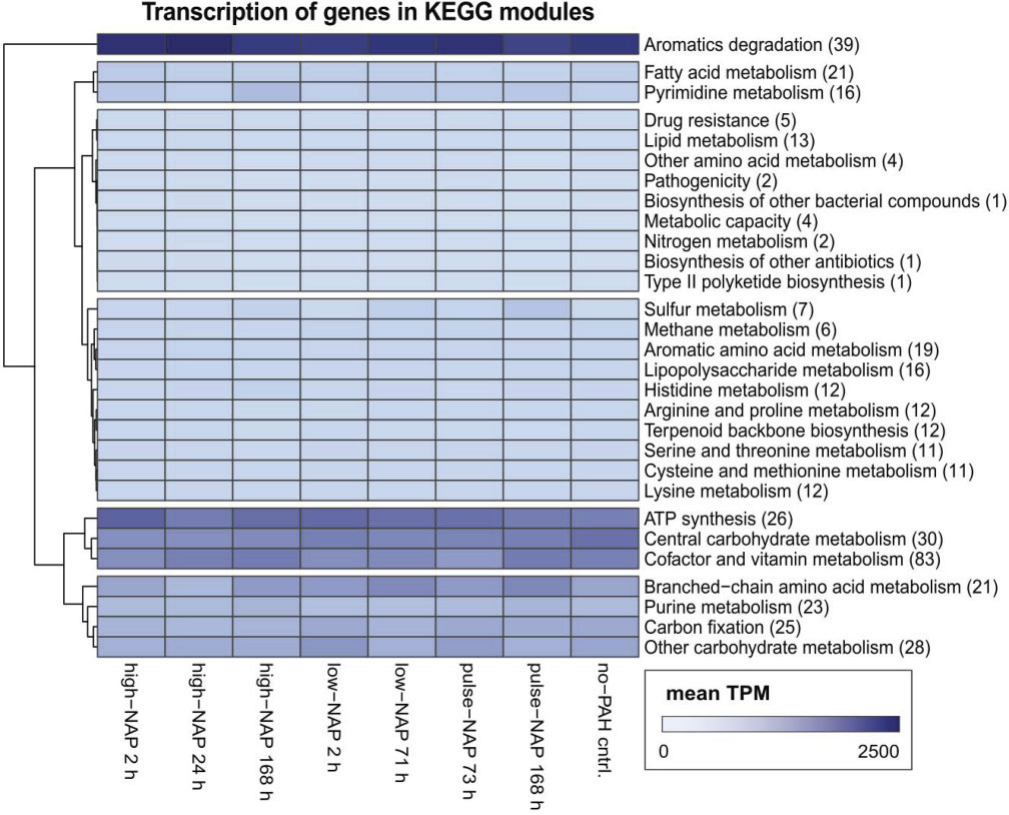
Expression per set of genes accumulated in KEGG modules (level 3). The expression is given in mean TPM per biological triplicate: 0 (white) to 2500 (dark blue). The number of genes in each functional category is shown in brackets. High and low concentration experiments received 100 and 30 mg L^−1^ of naphthalene at T_0_, respectively, whereas the pulse treatments received 30 mg L^−1^ at T_0_ and after 71 h. Transcription was determined after 2, 24 and 168 h for high-NAP treatments, after 2 and 71 h for low-NAP treatments (i.e. right before pulsing) and after 73 and 168 h for pulse-NAP treatments. PAH-free controls with pyruvate as carbon equivalent were analyzed after 24 h (no-PAH).

To identify key metabolic functions, transcripts were grouped by functional categories. Among all transcripts, expression of genes related to aromatic hydrocarbon degradation was the highest of all KEGG modules (level 3) across treatments (even in the no-PAH control), confirming that PAH degradation is an important metabolic feature of *Cycloclasticus pugetii* strain PS-1 (Fig. 2). Notably, only 39 genes were annotated in the KEGG database as related to PAH degradation. However, we identified a set of 154 genes potentially involved in PAH degradation by using available databases and literature, indicating that there might be a larger group of genes related to PAH degradation, and the analysis based on KEGG modules might be underestimating the true activity of PAH-related genes in strain PS-1.

### Transcriptional response of genes on the PAH-gene cluster

**Figure 3.**
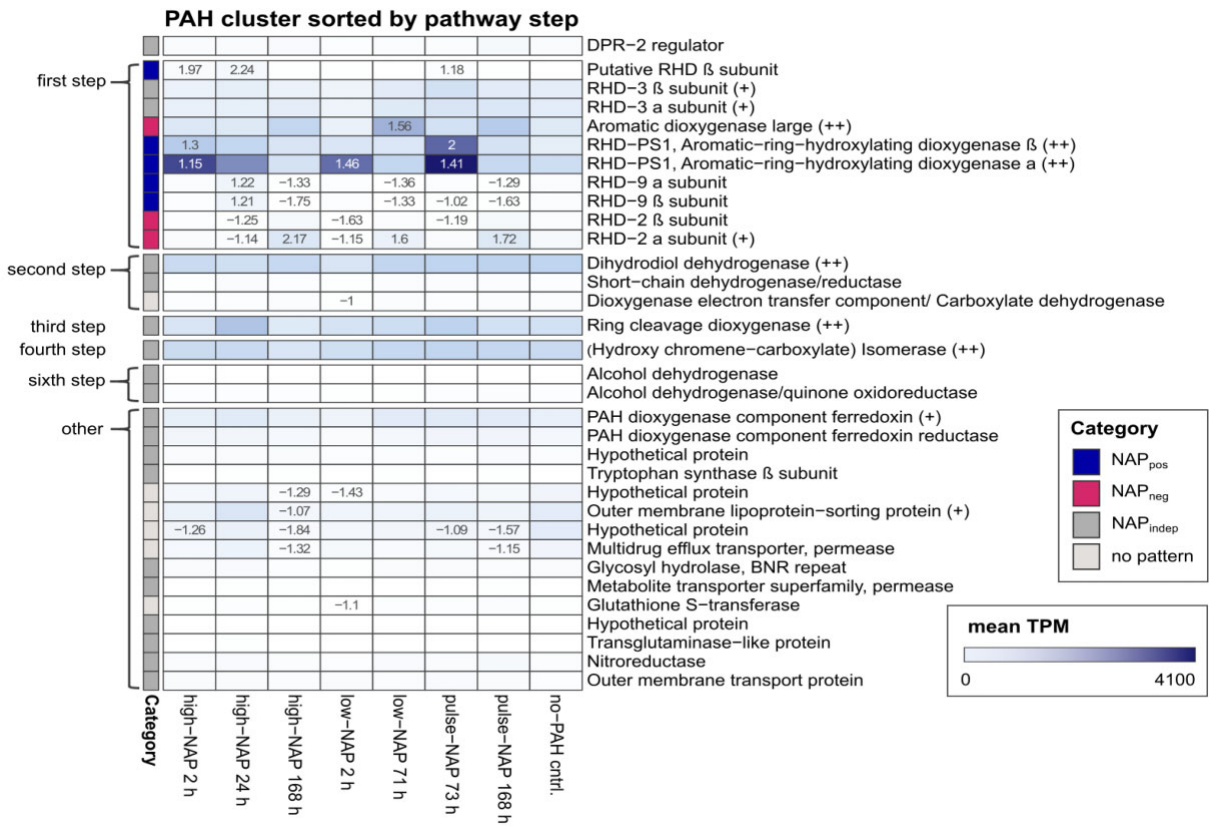
Expression in PAH cluster sorted by pathway step. Heatmap showing the expression (mean TPM per biological triplicate for each treatment) of genes associated with PAH degradation in the PAH cluster. Genes are sorted by pathway step (regulator, first step, second step, third step, fourth step, sixth step and other). Significant log2-fold changes to the no-PAH control are displayed (*P*_adj_ ≤ 0.05, 1 < log2-fold change > 1), 90th ≤ mean TPM_NAP_ < 95th percentile (+), and mean TPM_NAP_ ≥ 95th percentile (++) are indicated. High and low concentration experiments received 100 and 30 mg L^−1^ of naphthalene, respectively, at T_0_, whereas the pulse treatments received 30 mg L^−1^ at T_0_ and after 71 h. Transcription was determined after 2, 24 and 168 h for high-NAP treatments, after 2 and 71 h for low-NAP treatments (right before pulsing) and after 73 and 168 h for pulse-NAP treatments. PAH-free controls with pyruvate as carbon equivalent were measured after 24 h.

To further explore the transcriptional activity of *Cycloclasticus pugetii* strain PS-1 during PAH degradation, we examined the expression of a cluster of 33 genes, some of which were annotated in KEGG and all of which were previously identified as PAH-degradation genes in closely related *Cycloclasticus* spp. (Fig. 3, Table S3, sheet C, and Table S6) (Kasai et al. 2003, Wang et al. 2018). This gene cluster was previously described as “cluster E” and was differentially expressed in strain P1 grown on PAHs (naphthalene, phenanthrene and pyrene) compared with acetate-grown cells (Wang et al. 2018). Part of the cluster was published in 2003 as “*phnA*-cluster” (locus tag CYCPU_RS0111430 to CYCPU_RS0111480 in Table S6) and was described as enabling *Cycloclasticus* sp. strain A5 to degrade PAHs such as naphthalene and phenanthrene (Kasai et al. 2003). In the present study, the genes in the expression heatmap (Fig. 3) were sorted by pathway step of naphthalene degradation and significant transcription was highlighted [genes with −1 < log2-fold change < 1 and adjusted *P*-value (*P*_adj_) ≤ 0.05].

The full PAH-gene cluster in strain PS-1 contains nine genes that encode RHD subunits, including *rhd2α* and *rhd3α* [also referred to as *phnA1a* in previous publications (Kasai et al. 2003, McKew et al. 2007, Dionisi et al. 2011)]. The RHD subunits were identified as functional marker genes for PAH degradation, encoding enzymes that catalyze the first step of phenanthrene and naphthalene degradation, respectively (Wang et al. 2018). Further, CYCPU_RS0111455 was identified as putatively encoding an additional RHD β subunit based on its homology to other RHDs and the functional prediction of its active site (Paysan-Lafosse et al. 2023). Additional genes implicated in the PAH-degradation pathway were also identified in the gene cluster [e.g. a regulator factor (*dpr2*) (Wang et al. 2021)].

We used the significance (*P*_adj_) and expression (log2-fold) change to define categories based on the transcriptional response of a gene to the presence or absence of naphthalene. Of the 33 genes in the PAH cluster, five genes—four encoding RHDs and one for a putative RHD—were assigned to the NAP_pos_ category, meaning they were significantly upregulated in naphthalene-containing treatments and/or downregulated under naphthalene-starvation conditions compared with the no-PAH control. Conversely, three RHD-encoding genes were assigned to the NAP_neg_ category, demonstrating their lack of transcriptional upregulation in the presence of naphthalene and/or their upregulation under naphthalene-starvation conditions. A further 19 genes, like *rhd3α*, were naphthalene-independently expressed (NAP_indep_ category), and their transcription did not change over time, even in the presence of different naphthalene concentrations. Overall, only five of the 33 genes had low expression values, with a TPM below the 50th percentile (Fig. 3, percentile definition see Fig. S4), demonstrating the high expression trend of genes within the PAH cluster in strain PS-1. More importantly, the expression of another five genes was between the 90th and 95th percentile (mean TPM over all naphthalene-containing treatments between 451 and 878), and six were in the 95th percentile (mean TPM over all naphthalene-containing treatments equal and above 878), indicating very high expression (Fig. 3). These very highly expressed genes were annotated as encoding the small and large subunits of a RHD (CYCPU_RS0111490 and CYCPU_RS0111495, respectively), a dihydrodiol dehydrogenase/4-hydroxythreonine-4-phosphate dehydrogenase (CYCPU_RS0111480, second step of naphthalene degradation), a ring-cleavage dioxygenase (*rcd*) (CYCPU_RS0111460, third step of naphthalene degradation) and an (hydroxychromene-carboxylate) isomerase [CYCPU_RS0111430, fourth step of naphthalene degradation (Wang et al. 2018)]. The exceptionally highly expressed genes CYCPU_RS0111490 and CYCPU_RS0111495 are annotated to encode beta and alpha subunits of a RHD in the PAH cluster (Wang et al. 2018). However, the role of this RHD in naphthalene degradation is unknown as it remained untested in *Cycloclasticus* sp. strain P1 (Wang et al. 2018) and is uncharacterized in all other isolated *Cycloclasticus* sp. We, therefore, tentatively named them *rhdPS1β* and *rhdPS1α*, respectively. Using the basic local alignment search tool—BLAST (Altschul et al. 1990, Zhang et al. 2000, Morgulis et al. 2008)—we confirmed that the genes with the highest nucleotide pairwise identity were aromatic ring-hydroxylating dioxygenase subunits alpha and beta from *Cycloclasticus* sp. strain P1 and *Cycloclasticus zancles* 78-ME with 98.01% and 99.79% for *rhdPS1α* and *rhdPS1β*, respectively. Protein functional analysis with InterPro (Paysan-Lafosse et al. 2023) confirmed that the genes encoded RHD alpha and beta subunits and that *rhdPS1α* contains the Rieske [2Fe-2S] iron-sulphur domain. In the KO database (Kanehisa and Goto 2000, Kanehisa et al. 2016a,b), *rhdPS1α* and *rhdPS1β* are listed (#K16320) as involved in aminobenzoate degradation. Current literature has investigated potentially similar genes in a *Sphingomonas* sp. and a *Burkholderia* sp. (Chang et al. 2003, Gai et al. 2010); however, the respective genes are not highly related: the nucleotide pairwise identities were 48.1% and 55.5% for *rhdPS1α* and 49.3% and 50.2% for *rhdPS1β*, respectively. The transcription of two other RHD-encoding genes in the PAH cluster (*rhd9α*—CYCPU_RS0111505 and *rhd9β*—CYCPU_RS0111510) responded positively to naphthalene availability. The mean TPM over all naphthalene-containing treatments for *rhd9α* and *rhd9β*, however, was between the 50th and 75th percentile, indicating lower expression and a potentially lesser role during naphthalene degradation. Two additional genes encoding for RHD subunits (*rhd3α*—CYCPU_RS0111470 and *rhd3β*—CYCPU_RS0111465) were expressed independently of naphthalene availability, while three genes (*rhd2α*—CYCPU_RS0111555, *rhd2β*—CYCPU_RS0111560 and *phnA1b*—CYCPU_RS0111475) were assigned to the NAP_neg_ category.

We identified genes encoding all the enzymes prior to step 6 of the naphthalene-degradation pathway (Wang et al. 2018), except for a hydratase aldolase, which is necessary for the fifth step. However, the two genes encoding for two alcohol dehydrogenases (sixth step) were not highly expressed (i.e. CYCPU_RS0111520 mean TPM_NAP_ ≤ 50th percentile and CYCPU_RS0111545 mean TPM_NAP_ between the 50th and 75th percentile), indicating that some PAH-related genes used in naphthalene degradation by strain PS-1 may be located elsewhere in the genome.

From further analysis of the significantly upregulated and downregulated genes between the high-NAP-2 h and the no-PAH control, we identified an additional 184 genes that were differentially expressed (Fig. S5). In total, 23 of those genes (Fig. S5–marked as black stars) were found in the 154 PAH-related gene-database compiled from the literature (Table S3, sheet A), and only four of these 23 were part of the previously described PAH cluster (Fig. 3, Table S3, sheet C, and Table S6), indicating that manual curation of genes involved in PAH degradation is essential.

### Transcription of genes highly involved in PAH degradation

**Figure 4.**
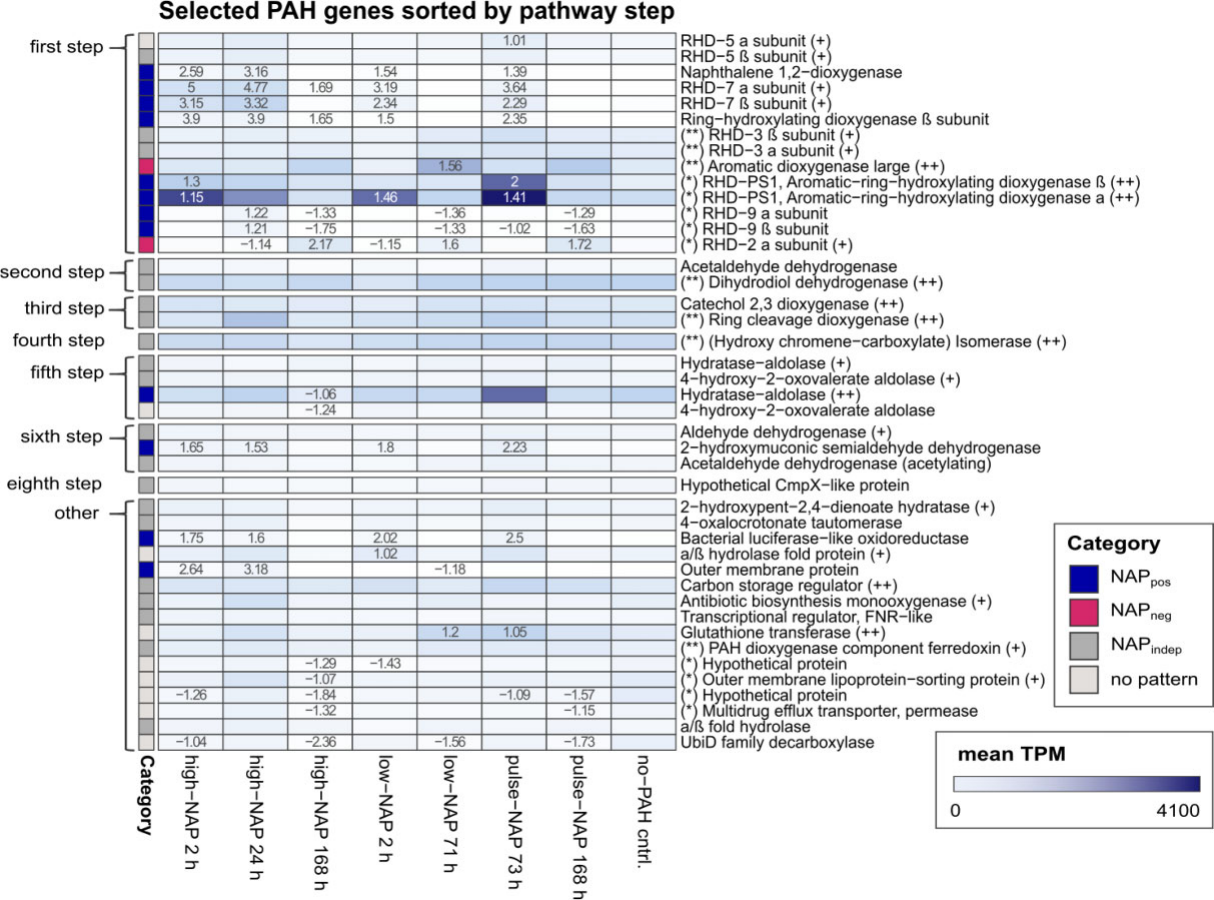
Selected, highly expressed PAH degradation genes. Heatmap showing the expression: mean TPM from 0 (white) to 4100 (dark blue) per biological triplicate. Genes were selected if TPM was within the 90th percentile in at least one naphthalene-containing treatment. 90th ≤ mean TPM_NAP_ < 95th percentile (+), and mean TPM_NAP_ ≥ 95th percentile (++) are indicated. Significant log2-fold changes compared with the control (pyr.24 h) are displayed (*P*_adj_ ≤ 0.05, 1 < log2-fold change > 1). Genes are sorted by pathway step (from top to bottom: first step, second step, third step, fourth step, fifth step, sixth step, eighth step and others). High- and low-concentration experiments received 100 and 30 mg L^−1^ of naphthalene, respectively, at T_0_, whereas the pulse treatments received 30 mg L^−1^ at T_0_ and after 71 h. Transcription was determined after 2, 24 and 168 h for high-NAP treatments, after 2 and 71 h for low-NAP treatments (i.e. right before pulsing), as well as at 73 and 168 h for pulse-NAP treatments. PAH-free controls with pyruvate as carbon equivalent were measured at 24 h. Genes that are part of the PAH-cluster are indicated [W Wang et al. (2018) (*), Kasai et al. (2003) (**)].

To identify genes for all the reactions actively involved in the naphthalene-degradation pathway, a subset of genes that were highly expressed in the presence of naphthalene (i.e. TPM ≥ 90th percentile in at least one of the naphthalene-containing treatments) was selected from the curated literature database, containing 154 PAH-related genes, and further analyzed (Table S3, sheets A and B). Out of this subset of 43 selected genes (Fig. 4, Table S3, sheet B), 16 genes were already known as part of the previously described PAH cluster (Fig. S6). From these 43 genes, 12 fell into the NAP_pos_ category (Fig. S6) and eight of those were annotated as encoding for RHD subunits. The only two genes in the NAP_neg_ category (both already known from the PAH cluster, see Fig. S6) also encoded RHD subunits. Most of the remaining genes were expressed independently of naphthalene concentration (20 genes of the selection, three of them encoding RHD subunits, Fig. S6), while the final nine genes showed no clear naphthalene-related pattern of expression. Further genes were potentially relevant for the strain due to their high expression (15 genes—90th ≤ mean TPM_NAP_ < 95th percentile) or very high expression (10 genes—mean TPM_NAP_ ≥ 95th percentile). Although 14 genes, which were not part of the PAH cluster, were identified as highly expressed (90th ≤ mean TPM_NAP_ < 95th percentile) and four as very highly expressed (mean TPM_NAP_ ≥ 95th percentile), the genes with the highest TPM values were still *rhdPS1α*, followed by *rhdPS1β*, which fell into the NAP_pos_ category. Additionally, a gene encoding a hydratase aldolase (CYCPU_RS0105800) that could potentially conduct the fifth step in the naphthalene-degradation pathway (Fig. S1) had very high expression and was in the NAP_pos_ category. Overall, 25 out of the selected 43 genes could be assigned to one of the steps from 1 to 7 (Fig. S1) in the naphthalene-degradation pathway, and 14 of those genes coded for RHD subunits, potentially involved in the initial step of the degradation pathway (Fig. 4).

## Discussion

### Naphthalene-dependent transcription of functional marker genes

**Figure 5.**
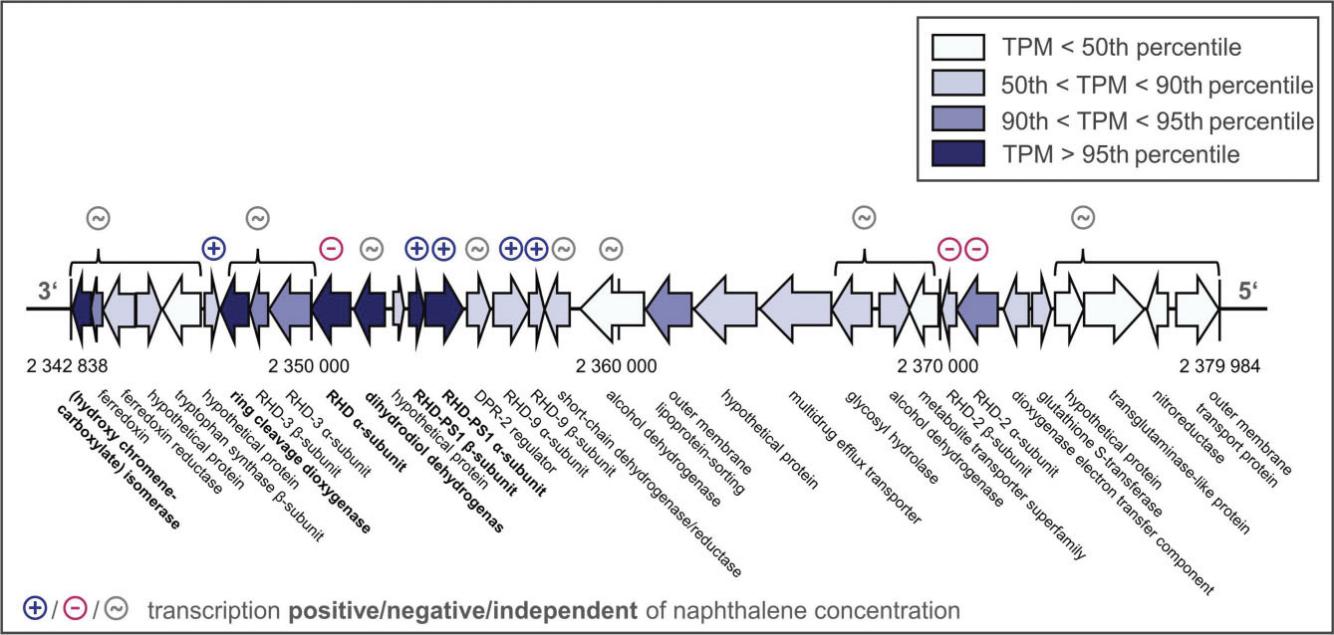
Gene cluster of PAH degradation-related genes within the *Cycloclasticus pugetii* PS-1 genome. Further information on the genes is provided in Table S3, sheet C, and Table S6; the cluster was previously described in closely related *Cycloclasticus* sp. strains A5 and P1 (Kasai et al. 2003, W Wang et al. 2018). Coloring indicates the percentile of expression level by mean TPM values over all naphthalene-containing experiments in our study (Fig. S4). Color coding for naphthalene-concentration dependence: blue, NAP_pos_; pink, NAP_neg_; gray, NAP_indep_. PAH cluster spanning 37 146 bp; total length of the genome is 2 383 924 bp.

We identified genes encoding an uncharacterized RHD alpha and beta subunit (termed *rhdPS1α* and *rhdPS1β*) whose expression responded significantly to naphthalene (Fig. S1). Although located in the previously described PAH cluster, as illustrated in Fig. 5 (Kasai et al. 2003, Wang et al. 2018), RHD-PS1 has not been characterized in any *Cycloclasticus* species to date. Comparing the transcription with the expression of other RHD-encoding genes, the substantial expression of *rhdPS1α* and *rhdPS1β* in response to naphthalene availability and degradation activity of *Cycloclasticus pugetii* strain PS-1 suggests that the RHD-PS1 dominates the first step of naphthalene degradation. The newly described genes are, therefore, promising candidates for functional marker genes and could potentially be used for monitoring the naphthalene-degradation activity of strain PS-1 with a qPCR-based method that quantifies the transcript-to-gene ratio (Baelum et al. 2008, Brow et al. 2013, Tentori and Richardson 2020, Vogel et al. 2023b), and could ultimately help to track PAH-degradation activity in contaminated environments. Knockout mutant (Perez-Pantoja et al. 2009) as well as recombinant-protein-expression studies (Wang et al. 2018) would help to further determine the role of *rhdPS1α* and *rhdPS1β* in the naphthalene-degradation pathway of *Cycloclasticus pugetii* strain PS-1.

Additionally, we identified four genes encoding RHD alpha and beta subunits that responded positively to naphthalene input and are likely involved in the first step of naphthalene degradation (Fig. 4). Two of the genes—*rhd7α* (CYCPU_RS0104890) and *rhd7β* (CYCPU_RS_0 104 895)—were highly expressed (90th ≤ mean TPM_NAP_ < 95th percentile) and the resulting RHD-7 was previously described as an enzyme that initializes fluoranthene degradation in *Cycloclasticus* sp. strain P1 (Wang et al. 2018). Further studies are needed to confirm the role of these other NAP_pos_ RHDs in naphthalene degradation, however, the observed link between transcription and naphthalene-degradation activity indicates they may play a key role in *Cycloclasticus pugetii* strain PS-1. The highly, and naphthalene-dependent, expressed genes might enable strain PS-1 to rapidly increase the number of enzymes (e.g. RHD-PS1 and RHD-7) and thereby quickly adapt to acute naphthalene contamination.

The transcription of three additional RHD-encoding genes among the PAH cluster (Fig. 5) depended significantly on the available naphthalene concentration. However, the genes were downregulated in the presence and upregulated in the absence of naphthalene, so they were attributed to the NAP_neg_ category. While *rhd2β* was highly expressed (90th ≤ mean TPM_NAP_ < 95th percentile) in the absence of naphthalene, we observed the substrate-independent transcription of *rhd2α* in previous growth experiments with naphthalene and phenanthrene (Vogel et al. 2023b). This could indicate that transcription of *rhd2α* and *rhd2β* are not only dependent on the availability of naphthalene, but also the cultivation conditions of the organism (growth experiment vs. cell-suspension experiment) or as a response to starvation (Vogel et al. 2023b). Notably, these RHD-encoding genes might exhibit a different transcriptional behavior for alternative PAHs and further studies should examine the transcription of the identified marker genes in response to other PAH substrates such as phenanthrene, biphenyl and naphthalene derivates.

### Substrate-independent transcription of PAH genes

No significant upregulation in naphthalene-rich and/or downregulation under naphthalene-starvation conditions compared with the no-PAH controls indicated transcription independent of naphthalene availability (NAP_indep_ category).

Two of those genes were encoding the ferredoxin and ferredoxin reductase (part of the PAH cluster, Fig. 5), which are important parts of the multicomponent RHD enzymes, and thus, relevant in the first step of PAH degradation. However, the resulting components are often shared between different RHD enzymes (Wang et al. 2018). Given this, it is not surprising to find the genes as constitutively or naphthalene-independently expressed in an organism that has highly expressed genes for at least six different RHDs.

Out of 13 genes potentially encoding enzymes involved in the naphthalene-degradation steps 2 to 6 (Fig. S1)—from the entire genome—10 were naphthalene-independently expressed, indicating that substrate-independent expression of genes in the PAH degradation pathway occurs regularly in strain PS-1. Although all the genes involved in the downstream steps are likely associated with PAH degradation, some of them could produce enzymes that are involved in other pathways (Hernaez et al. 2002). Overall, it remains uncertain if the isomerases, dehydrogenases and hydratase aldolases are specific for PAH degradation. The non-exclusive use of the downstream enzymes in other metabolic pathways might explain the naphthalene-independent expression of some of the PAH-degradation genes; however, further investigation would be required to prove this hypothesis.

RHDs, however, are specific for the first step in PAH degradation (Gibson and Parales 2000, Singleton et al. 2012, Yesankar et al. 2023). Finding three genes encoding RHD subunits that are highly expressed (90th ≤ mean TPM_NAP_ < 95th percentile) and naphthalene independent is, therefore, surprising. The genes *rhd3α, rhd3β* and *rhd5β* all fell into the NAP_indep_ category, and while their highly expressed nature makes them potentially important for strain PS-1, their transcription is likely not a response to acute naphthalene input. These results were corroborated in previous qPCR-based experiments where substrate-independent transcription was observed for *rhd3α* as well as for *pahE* (a hydratase aldolase; CYCPU_RS0105800) in *Cycloclasticus pugetii* strain PS-1 (Vogel et al. 2023b).

The reasons for substrate-independent transcription of PAH-degradation genes, especially RHDs, are unknown. Given that PAH degradation is central to the metabolism of *Cycloclasticus pugetii* strain PS-1, as shown by analyzing the transcription per functional category (Fig. 2), the corresponding enzymes might be essential for the lifestyle of this highly specialized organism. Constitutive—i.e. substrate-independent—expression of functional genes has been observed in other hydrocarbon-degrading bacteria (Cunliffe et al. 2006, Churchill et al. 2008), making it a potentially common—but overlooked—phenomenon. Further, substrate-independent transcription of RHD-encoding genes could have practical reasons for this organism. Due to the PAH-independent expression of *rhd3α, rhd3β, rhd5β* and potentially *rhd5α*, RHD-3 and RHD-5 might be used as a “background” naphthalene-degradation system for strain PS-1. The PAH-independent expression should allow a constant availability of these enzymes, which might lead to substrate-independent PAH-degradation capacity.

### Genetic redundancy in the PAH-degradation pathway of *Cycloclasticus pugetii* strain PS-1

Genetic redundancy is a common phenomenon in all pathway steps, but particularly in the first step of naphthalene degradation. Five RHDs (RHD-PS1, RHD-7, RHD-3, RHD-5 and RHD-2) were identified, for which transcription of at least one subunit-encoding gene was within the 90th percentile.

The reasons for genetic redundancy in PAH-degradation genes, especially in genes encoding RHDs, are unknown. RHDs catalyze the first reaction, which is the rate-limiting step in PAH-degradation, so the rate of carbon and energy gain increases with an accelerated rate of the first step. Potentially, genetic redundancy of RHD-encoding genes could be beneficial for strain PS-1 by providing several enzymes for the same function and, thereby, increasing the rate of the first step in naphthalene degradation (Perez-Pantoja et al. 2009). Additionally, a fast consumption of naphthalene should lead to a steeper gradient between the bioavailable dissolved naphthalene and the pure compound. The steeper gradient in turn would accelerate the dissolution rate of naphthalene (Volkering et al. 1992, 1993, Vogel et al. 2023a), leading to a faster substrate supply rate for the organism.

Genetic redundancy of RHD-encoding genes could, alternatively, indicate a highly specialized PAH-degradation strategy of *Cycloclasticus pugetii* strain PS-1. Potentially, two PAH-degradation enzymatic systems could be used, depending on the availability of PAHs: a steadily available “background” system and a specialized “rapid-response” system for acute PAH input. The “background” system would include RHDs that were encoded by substrate-independently expressed genes that would be constantly available and could degrade chronic PAH contamination at trace amounts. Considering that most genes encoding these naphthalene-degradation-pathway enzymes were expressed independently of naphthalene addition (NAP_indep_ category), it is reasonable to assume that the substrate-independent expression of highly relevant PAH-degradation genes is the default strategy for *Cycloclasticus pugetii* strain SP-1. Moreover, all of the pathway steps other than the first step were shared for multiple PAHs (i.e. naphthalene, phenanthrene and pyrene) in a closely related *Cycloclasticus* sp. (Wang et al. 2018), which emphasizes how essential those genes are in this potential “background” system. The “rapid-response” system, on the other hand, would include (mainly) RHDs that were upregulated and downregulated depending on the availability of substrates and would be expressed in the case of a high-contamination event to quickly degrade substrates at high concentrations. This system would enable *Cycloclasticus pugetii* strain PS-1 to respond quickly and benefit from the sudden availability of high loads of substrate. Moreover, the enzymes encoded by genes in this system might be more substrate specific because they potentially only need to respond to one or two PAHs and convert them at high rates. RHD-3 and RHD-5 would, therefore, potentially serve as a “background” metabolism, which could be active in naphthalene- or PAH-free environments, whereas RHD-PS1 and RHD-7 might be responding to acute naphthalene input.

### *Cycloclasticus* spp. in the environment

The hypothesized PAH-degradation strategy of *Cycloclasticus pugetii* strain PS-1 offers several potential benefits to the growth of *Cycloclasticus* spp. in the environment. Environmentally occurring concentrations are much lower than those routinely used in the laboratory [e.g. the sum of dissolved concentrations of 64 PAHs in surface water samples was between 2 ng L^−1^ (Indian Ocean) and 3.5 ng L^−1^ (North Atlantic) (González-Gaya et al. 2016)]. Consequently, we hypothesize that *Cycloclasticus* spp., as part of a native community, are commonly operating with the “background” system and high-substrate affinity system of enzymes, for which the genes are substrate-independently expressed. Conversely, the “rapid-response” system, with enzymes encoded by PAH-dependently expressed genes, is likely triggered by higher substrate concentrations and consists of low-substrate affinity enzymes.

We propose that the “rapid response” system would be triggered by a naphthalene concentration lower than that of naphthalene solubility in seawater (28.96 mg L^−1^) given that environmental concentrations of naphthalene are, in reported cases, much lower than 28.96 mg L^−1^ (Diercks et al. 2010, González-Gaya et al. 2016, Vogel et al. 2023a). This “rapid response” enzymatic system could, for example, potentially have operated in the contaminated hydrocarbon plume that formed after the Deepwater horizon oil spill, where PAH concentrations amounted to a maximal 189 µg L^−1^ (Diercks et al. 2010). Changing the PAH/naphthalene input and thereby substrate availability, hence, is an important environmental condition that can influence the PAH/naphthalene-degradation rate considerably (Mostafa et al. 2019, Bacosa et al. 2021). Further studies should investigate the threshold concentrations required to activate the “rapid response” system.

Identifying genes of both enzymatic systems in our model organism *Cycloclasticus pugetii* strain PS-1 highlights the level of adaption to a PAH-degradation lifestyle. Considering *Cycloclasticus pugetii* strain PS-1 was isolated from Puget sound (Dyksterhouse et al. 1995)—a habitat with many natural oil seeps—a high level of adaption to both chronic traces of PAH, as well as recurring high-input PAH pollution, is not surprising. We anticipate that *Cycloclasticus* spp. can outcompete other PAH-degrading bacteria that only have one step or a subset of steps in the PAH-degradation pathway. The advantage of the *Cycloclasticus* spp. would arise from them having multiple genes for each step of the pathway and hypothetically a “rapid-response” system of (mainly) RHDs (Sieradzki et al. 2021). The substrate-dependent transcription of (mainly) RHDs might give *Cycloclasticus pugetii* strain PS-1 the possibility to rapidly increase its degradation rate, which could be one explanation why *Cycloclasticus* spp. are detected ubiquitously and can be isolated from PAH-amended enrichment cultures (Wang et al. 2008, Gutierrez et al. 2013, Cui et al. 2014, Rizzo et al. 2019).

### Environmental implications

With the increasing amount of environmental metagenomic and metatranscriptomic studies, it is important to consider what the detection of genes and transcripts from the NAP_pos_ and NAP_indep_ categories would imply for the respective microbial communities and environments. The detection of genes from the NAP_indep_ category, such as *rhd3α* and *rhd3β*, or genes encoding downstream enzymes, such as *pahE*, could be—given that the genes are truly PAH-pathway specific—used as marker genes for PAH-degrading organisms, as previously suggested (Dionisi et al. 2011, Liang et al. 2019). The transcription of those genes, however, does not imply that the organisms are actively degrading PAHs, but rather indicates a general metabolic activity. Further, an environment with microbial community members possessing NAP_indep_ genes may not always be pristine (Gutierrez 2019b), even if no apparent PAH contamination is detectable (Angelova et al. 2021). We, therefore, posit that some environments may be continually purged from constant trace inputs of PAHs without enriching PAH degraders or inducing PAH-degrading pathways, given that a “background” system of genes is constantly transcribed.

By contrast, genes from the NAP_pos_ category, especially *rhdPS1α* and *rhdPS1β*, could potentially not only identify PAH-degrading organisms, but also be used as functional marker genes for high naphthalene-degradation activity of *Cycloclasticus pugetii* strain PS-1. Determining the transcript-to-gene ratio of NAP_pos_ genes of a microbial community could be a valuable tool to quantify the cell-number-independent degradation rate of specific compounds and, thereby, assess the PAH-biodegradation performance after high-concentration contamination events, like oil spills or in a laboratory experiment (Wilson et al. 1999, Baelum et al. 2008, Brow et al. 2013, Tentori and Richardson 2020, Vogel et al. 2023b). Further, identifying organisms with NAP_pos_ genes in an environmental community could indicate a faster environmental recovery from acute high-input contamination.

Nonetheless, genetic redundancy in key genes for PAH degradation makes it difficult to quantify the PAH-degradation activity in environments based on the expression of a single gene or set of genes. Detailed knowledge about the PAH-degrading community and the categorization of the involved PAH-degradation genes would be necessary to select a suitable set of target genes for the investigated environment. Moreover, the expression of such identified target genes could vary between strains and could be sensitive to other environmental factors. Further studies like a global assessment and characterization of RHDs in all currently available metagenomes and transcriptomes are necessary before a robust set of genes for the quantification of *in situ* PAH-degradation rates can be proposed.

### Open questions on genetic redundancy in PAH degradation

Several open questions remain regarding genetic redundancy in *Cycloclasticus pugetii* strain PS-1. Further studies, including knockout mutants and enzymatic assays, are required to investigate for molecular redundancy (Perez-Pantonja et al. 2009), identify which of the RHDs have an affinity for other PAHs, or to confirm these RHDs are performing the initial step in naphthalene degradation (Wang et al. 2018). Additional research is necessary to determine whether the expressed RHD-encoding genes are induced by naphthalene, but the corresponding enzymes are not produced and/or not used in naphthalene degradation.

Moreover, in a closely related *Cycloclasticus* sp., the genes encoding RHD-2, RHD-3 and five other genes associated with the PAH-degradation pathway were co-regulated by the same regulator (Table S6) (Wang et al. 2021). In *Cycloclasticus pugetii* strain PS-1, however, these genes were expressed differently: some NAP_neg_ and others were substrate independent. Our understanding of conditions and substrates influencing the regulation of PAH-degradation genes in *Cycloclasticus* spp. remains undefined. Further studies are needed to determine if an alternative regulation mechanism is used in *Cycloclasticus pugetii* strain PS-1, or if the genes are co-regulated but the mRNA of the apparent NAP_neg_ genes is potentially degraded and, therefore, not substrate-independently detected.

Because the model organism in this study is an isolated and very well studied *Cycloclasticus* sp., further studies could investigate if the observed transcriptional patterns changed in the case when *Cycloclasticus pugetii* strain PS-1 would live as part of a PAH-degrading community. Further, Arctic *Cycloclasticus* spp. from a natural community were shown to have a different set of RHDs than expected from the genomes of the isolated *Cycloclasticus* spp. (Vogel et al. 2023b). Investigating the transcription of the PAH genes in environmental *Cycloclasticus* spp. under PAH-free or even hydrocarbon-free conditions—given that some *Cycloclasticus* spp. can degrade alkanes (Rubin-Blum et al. 2017, Gutierrez et al. 2018)—would be the next step. Because the cell numbers of *Cycloclasticus* spp. under hydrocarbon-free conditions are typically low and mostly not detectable, assessing the transcription of PAH-degradation genes will be challenging. Therefore, quantifying transcripts (through qPCR) or conducting metatranscriptomic studies following an environmental contamination event when all hydrocarbons are consumed, similar to our starvation conditions, could elucidate the role of *Cycloclasticus* spp. in an environmental microbial community.

## Conclusion

The naphthalene-dependent transcription of multiple RHDs indicated that strain PS-1 is very well adapted to respond instantly to high-concentration inputs of PAHs. This fast reaction can potentially be achieved by increasing the overall degradation rate through the maintenance of an enzymatic “rapid response” system. Currently, it is not possible to deduce the degraded PAH or the environmental degradation activity by targeting the transcription of a single functional gene or set of genes. The newly described functional marker genes *rhdPS1α* and *rhdPS1β*, however, are promising target-gene candidates to quantify naphthalene-degradation activities through DNA/RNA-based methods, because their transcription seems to correlate to naphthalene degradation in *Cycloclasticus pugetii* strain PS-1. Using these genes, the monitoring of PAH degradation could, in future, be conducted in a high-throughput manner by using molecular-based methods such as the TtG ratio. This in turn could facilitate more efficient PAH bioremediation because the measures or conditions could be adapted more rapidly when the monitoring proves that the degradation rate is changing. Further, an additional set of PAH-degradation genes that were expressed independently of naphthalene was also identified. Those genes are involved in all reactions of the currently known naphthalene-degradation pathway in *Cycloclasticus* spp., indicating there might be another set of PAH-degrading enzymes that is potentially used as a “background” system for the degradation of environmentally occurring trace amounts of PAHs.

The observed genetic redundancy in PAH-related genes—particularly RHDs—along the naphthalene-degradation pathway and their varying levels of transcription under different conditions has not been reported previously and should be further studied. This genetic flexibility indicated by the hypothesized two enzymatic systems could enable PAH degraders to respond to fluctuating hydrocarbon inputs in a need-based way. Understanding the degradation pathway used by key PAH degraders under varying conditions, such as low vs. high PAH concentrations, is important to assess contamination scenarios correctly. These assessments could be used to enhance bacterial PAH degradation in, for example, bioremediation scenarios.

## Supplementary Material

fiae060_Supplemental_Files

## Data Availability

The raw RNA-sequencing data from this study have been deposited with links to the BioProject accession number PRJNA838751 into the NCBI BioProject database (https://www.ncbi.nlm.nih.gov/bioproject/PRJNA838751).
