# Supplementary material for: Genetic redundancy in the naphthalene-degradation pathway of Cycloclasticus pugetii strain PS-1 enables response to varying substrate concentrations
Source: FEMS Microbiol Ecol. 2024 Apr 13;100(6):fiae060. doi: 10.1093/femsec/fiae060 (PMC11099662; doi:10.1093/femsec/fiae060)
Supplement: fiae060_Supplemental_Files [file fiae060_supplemental_files.zip › Vogel_2023_Supporting data_Information_Genetic_redundancy_in_naphthalene-degradation_pathway (1).pdf]

Genetic redundancy in the naphthalene-degradation pathway of *Cycloclasticus pugetii* strain PS-1 enables response to varying substrate concentrations

Anjela L. Vogel, Katharine J. Thompson, Daniel Straub, Florin Musat, Tony Gutierrez,

Sara Kleindienst

Supporting Information

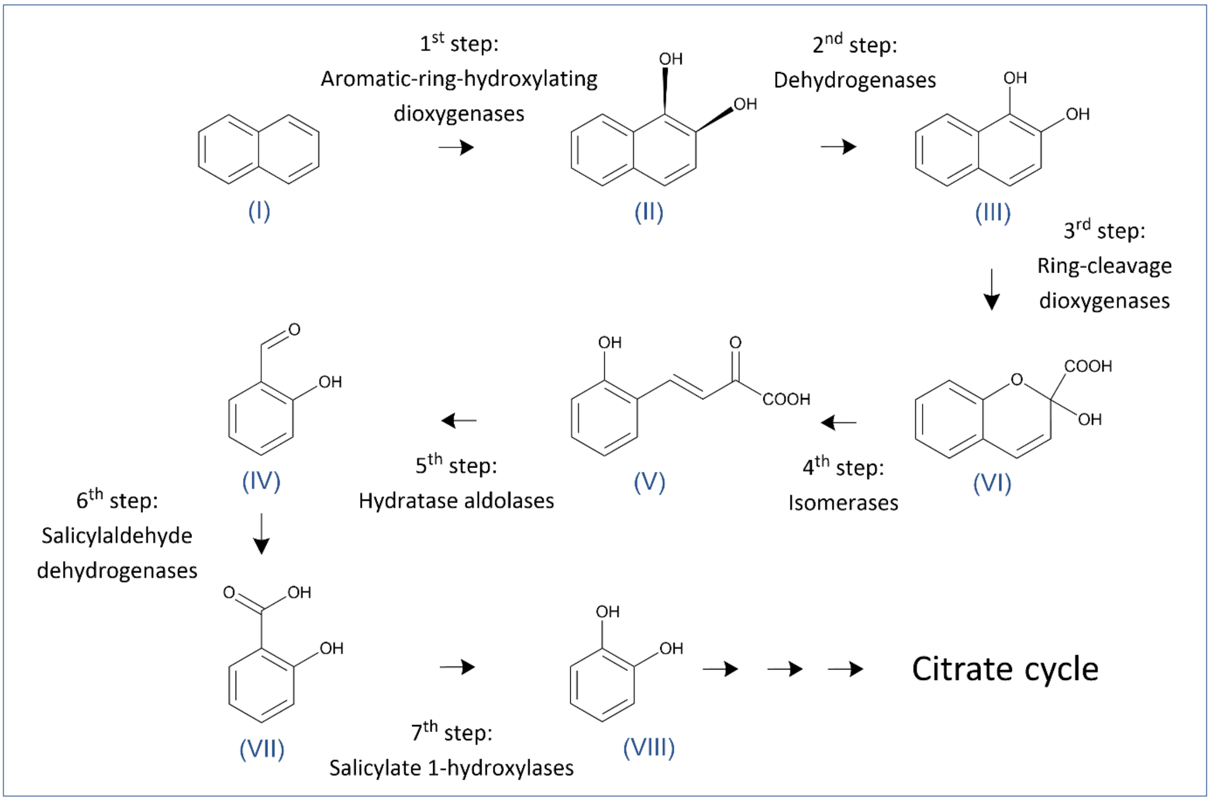

Fig. S1: Enzymes (labelled in the figure) and molecules involved in the biochemical naphthalene-degradation pathway in *Cycloclasticus spp.* (Wang *et al.*, 2018). (I) naphthalene, (II) *cis*-1,2-naphthalene-dihydrodiol, (III) 1,2-dihydroxynaphthalene, (IV) 2-hydroxy-2H-chromene-2-carboxylic acid, (V) *trans*-o-hydroxybenzylidene pyruvate, (VI) salicylaldehyde, (VII) salicylic acid, and (VIII) catechol.

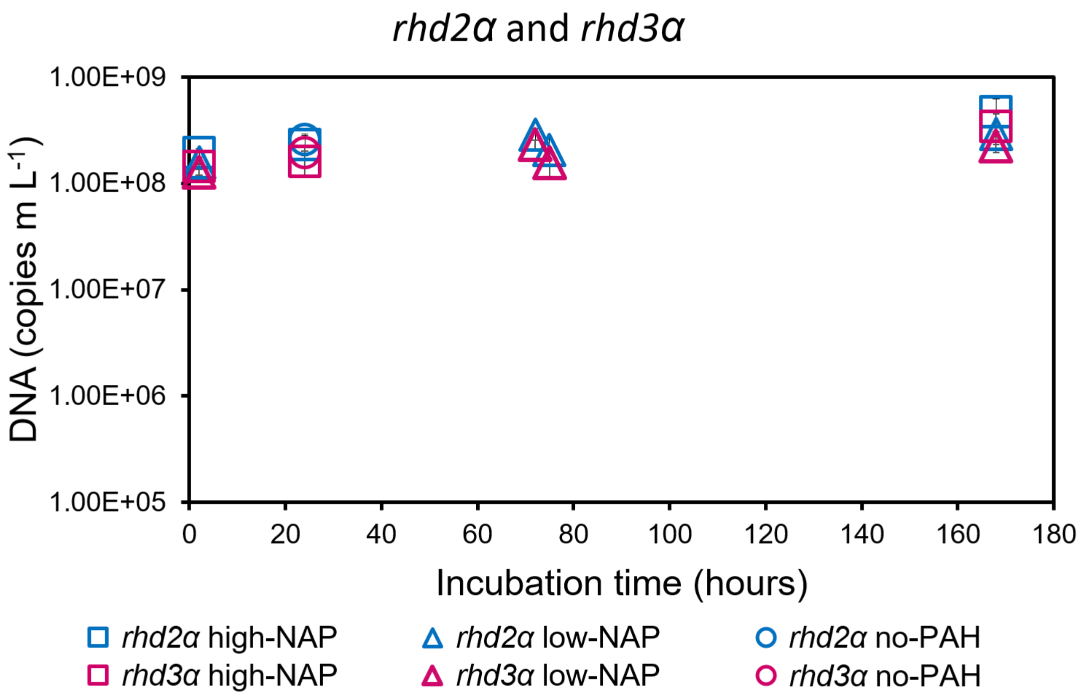

Fig. S2: Cell number estimates from DNA of two functional marker genes (*rhd2α* and *rhd3α* coloured blue and pink, respectively) in copies mL^{-1} quantified by qPCR in samples, which were selected for RNA sequencing. Incubation conditions were naphthalene at 100 mg L^{-1} and 30 mg L^{-1} (squares and triangles, respectively) as well as a PAH-free control, receiving pyruvate as carbon equivalent (circles). Error bars represent standard deviation between measured biological triplicates and are smaller than the markers.

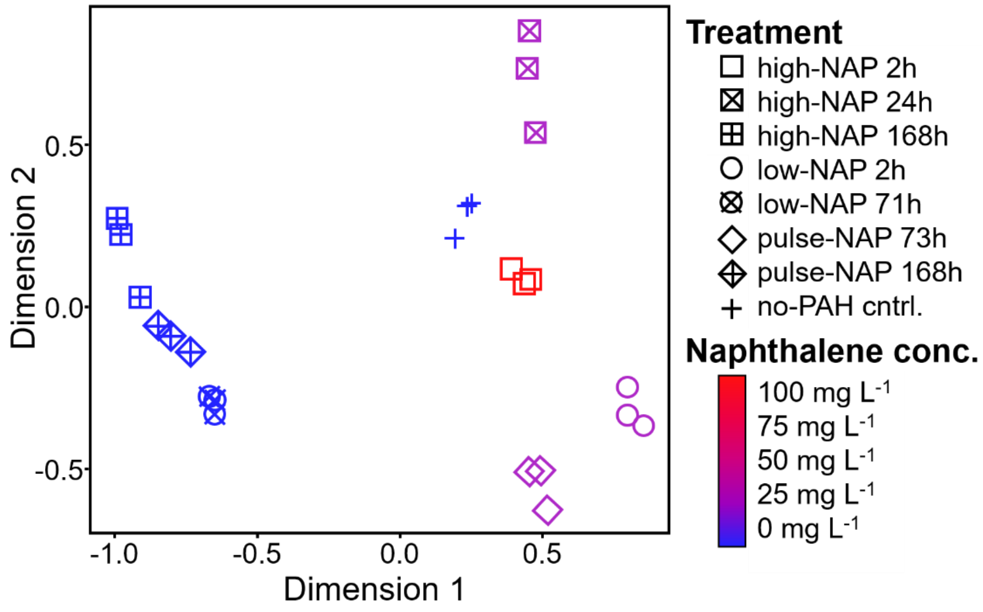

Fig. S3: Multidimensional scaling (MDS) plot of distances between gene expression profiles (all replicates displayed). Samples with similar gene expression are close to each other in the plot while samples that are dissimilar are further away from each other. Shapes indicate different treatments – high-NAP (squares), low-NAP (circles), pulse-NAP (diamonds) and no-PAH pyruvate control (cross). Color indicates naphthalene concentration high to low (red to blue).

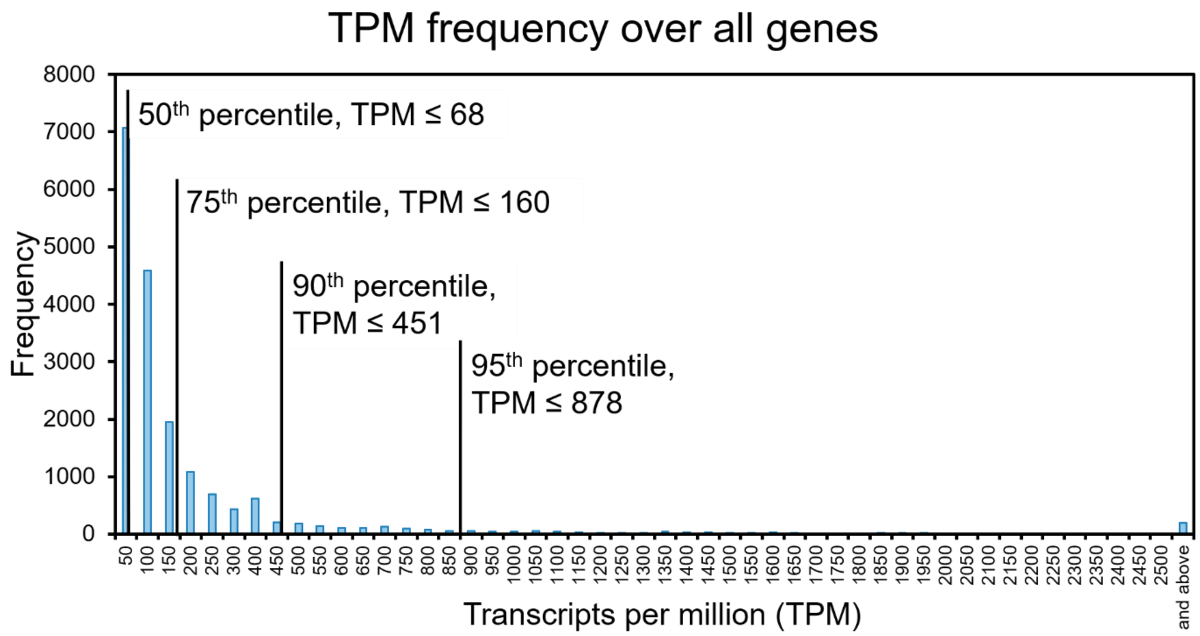

Fig. S4: Frequency of Transcripts per million (TPM) values of all genes over all treatments - 51 bins, 0 to 2500 in 50er bins. Marked 50th, 75th, 90th, and 95th percentiles were chosen to categorise expression levels, e.g. in Fig. 3,4, and 5.

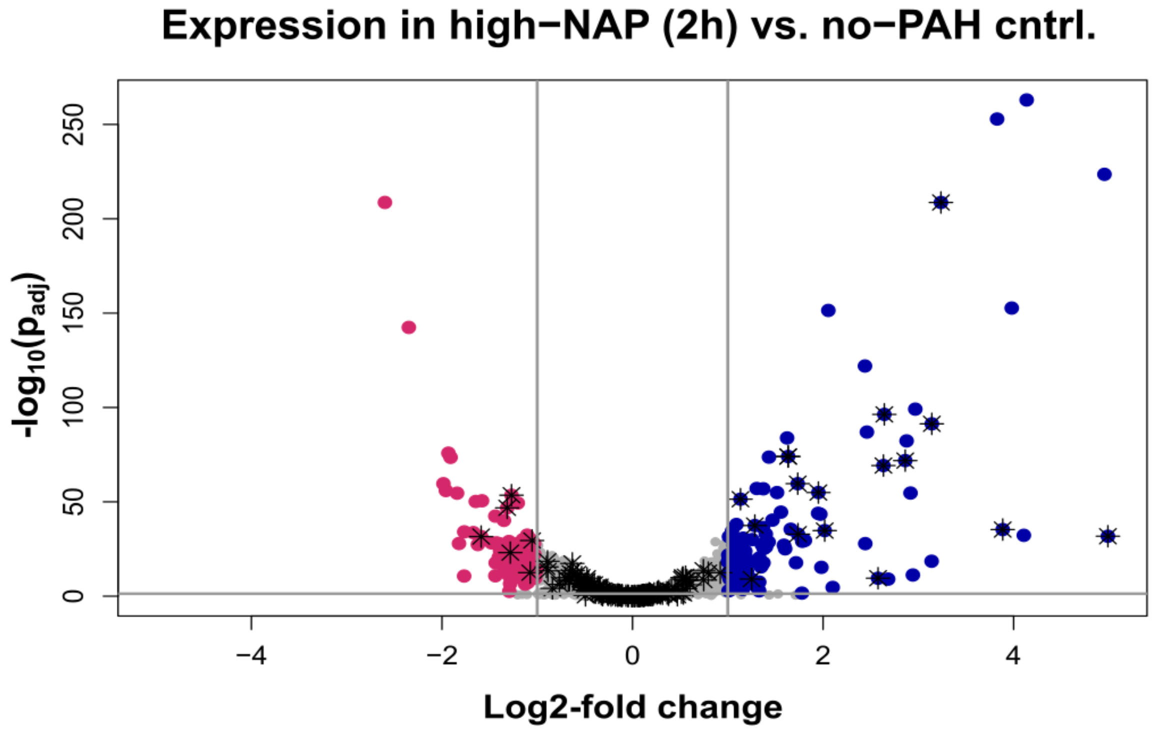

Fig. S5: Statistical significance (-log$_{10}$(p_{adj})) versus the magnitude of change (log2-fold change) of – high-NAP 2h versus the no-PAH pyruvate control (24 h). Means per biological triplicates are displayed, significance ($p_{adj} \leq 0.05$) and change (-1 < log2-fold change > 1) are indicated (pink and blue for down- and upregulation, respectively). 154 genes related to PAH-degradation curated from the literature are highlighted as black stars, also listed in Table S3, sheet A.

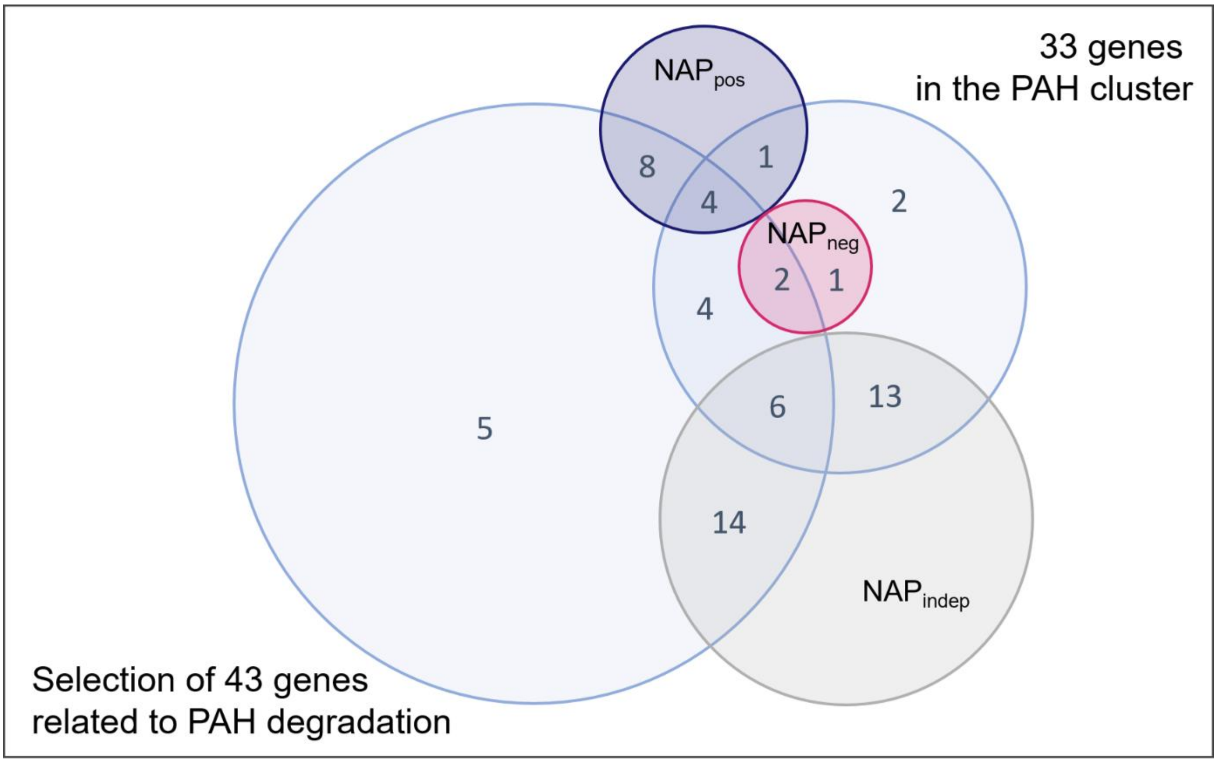

Fig. S6: Venn diagram illustrating intersection between subsets of investigated genes. The group of 43 genes (big light blue circle) was selected from the overall database containing 154 genes related to PAH degradation if TPM was within the 90th percentile in at least one naphthalene-containing treatment. The second main group represents the genes from a gene cluster previously associated with PAH degradation (Kasai *et al.*, 2003, Wang *et al.*, 2018). Small circles represent the categories with which the genes were associated based on their transcription in presence of naphthalene (see Table S4), NAP_{pos} (dark blue), NAP_{neg} (pink), and NAP_{indep} (grey). The remaining genes – 5 for the selection, 2 for the genes from the cluster, and 4 from both – fell into the "no pattern" category (not represented by a separate circle).

TABLE S1 Primer characteristics for functional marker genes *rhd2α* and *rhd3α*.

**Target gene**	**Primer name**	**Primer sequence 5' -> 3'**	**T_m [°C]**	**GC content [%]**	**Hairpin temp. [°C]**	**Primer-dimer temp. [°C]**	**qPCR product**
rhd2α[1,2]	rhd2α1126F	ACA CGA AGA GGA AAG CTG CA	59.9	50	x	1	199 bp
	rhd2α1305R	TTT TCT TGC CTG CAT AGC GC	59.8	50	42.9	x	
rhd3α[2,3]	rhd3α 669FD	GGG TGG ACT AGC TGG AA	54.8	59	x	3.2	120 bp
	rhd3α781RD	TTC GCA TGA ATA GCG ATG G	55.9	47	59.1	11.2	

[1]Primer design from our previous study (Vogel *et al.*, 2023)
[2]Gene information (Wang *et al.*, 2018)
[3]Gene information (*rhd3α* is referred to as *phnA1*) and primer design from (Dionisi *et al.*, 2011)

TABLE S2 qPCR protocols for functional marker genes *rhd2α* and *rhd3α*. Volumes (10 µl in total) are given per reaction well.

	rhd2α[1,2]	***rhd3α***[2,3]
SYBER green	5 µL	5 µL
Primer F (0.5 µM)	1 µL	1 µL
Primer R (0.5 µM)	1 µL	1 µ
H_2O	2 µL	2 µL
Nucleotide tmpl.	1 µL	1 µL
Step 1	95°C, 5 min	95°C, 5 min
	1 cycle	*1 cycle*
Step 2 to 4	95°C, 20s	95°C, 20s
	62°C, 20s	62°C, 20s
		72°C, 20s
	40 cycles	*35 cycles*
Step 5	95°C, 1 min	95°C, 1 min
	1 cycle	*1 cycle*
Step 6	62°C, 30s	62°C, 30s
	1 cycle	*1 cycle*
Step 7	62°C – 95°C, 5s steps	62°C – 95°C, 5s steps
	melting curve	*melting curve*

[1]Primer design from our previous study (Vogel *et al.*, 2023)
[2]Gene information (Wang *et al.*, 2018)
[3]Gene information (*rhd3α* is referred to as *phnA1*) and primer design from (Dionisi *et al.*, 2011)

TABLE S3: Curated database of investigated genes related to PAH-degradation pathway in *Cycloclasticus pugetii* PS-1 with notes, references and expression data (mean TPM per biological triplicate) for each treatment. Sheet A) full set of 154 investigated genes, compiled using the annotations of the NCBI database (August 2022) (Schoch *et al.*, 2020), the KEGG database (August 2022) (Kanehisa & Goto, 2000, Kanehisa *et al.*, 2016, Kanehisa *et al.*, 2016), and published literature (Wang *et al.*, 1996, Kasai *et al.*, 2003, Wang *et al.*, 2018, Liang *et al.*, 2019, Wang *et al.*, 2021, Bagi *et al.*, 2022). Sheet B) genes selected if transcribed in naphthalene-containing treatments (mean TPM in at least one of the naphthalene-containing treatments within the 90th percentile, mean TPM ≥ 451) and sheet C) genes that are part of the previously investigated PAH-cluster (Kasai *et al.*, 2003, Wang *et al.*, 2018). **This table is available in an additional Microsoft Excel file.**

TABLE S4: Overview of the categorization of genes (categories NAP_{pos}, NAP_{neg}, NAP_{indep}, and no pattern) by transcriptional behaviour for each treatment. Transcription of the naphthalene-containing treatments is compared to the no-PAH controls and a gene is considered significantly up- (blue positive) or downregulated (pink negative) for $p_{adj} \leq 0.05$ and -1 < log2-fold change > 1, otherwise it is classified as PAH-independently expressed (grey indifferent). Genes are assigned to the NAP_{pos} and NAP_{neg} categories if the gene is up- or downregulated significantly in at least one of the treatments, even if some treatments show no change to the baseline.

	high-NAP 2 h	high-NAP 24 h	high-NAP 168 h	low-NAP 2 h	low-NAP 71 h	pulse-NAP 73 h	pulse-NAP 168 h	no-PAH cntrl.
**NAP_{pos}**	+	+	−	+	−	+	−	baseline
**NAP_{neg}**	−	−	+	−	+	−	+	baseline
**NAP_{indep}**	~	~	~	~	~	~	~	baseline
**no pattern**	irregular expression	irregular expression	irregular expression	irregular expression	irregular expression	irregular expression	irregular expression	baseline

Table S5: Naphthalene concentration in abiotic and pyruvate controls, measured by GC-MS. Samples were measured in duplicates. Error represents standard deviation.

Treatment	Time = 0 h	Time = 24 h	Time = 73 h	Time = 168 h
high-NAP	106.1±0.20 mg L^{-1}	n.a.	n.a.	100.8±0.78mg L^{-1}
low-NAP	31.39 ± 0.81 mg L^{-1}	n.a.	n.a.	31.32±0.67mg L^{-1}
pulse-NAP	n.a.	n.a.	59.08±1.31 mg L^{-1}	59.94±1.42 mg L^{-1}
No-PAH cntrl.	n.a.	0.00±0.0 mg L^{-1}	n.a.	n.a.

Table S6: Genes that are part of the PAH gene cluster in *Cycloclasticus pugetii* PS-1. Highly expressed genes (mean TPM over all naphthalene containing treatments > 95th percentile) are marked in bold. Log2-fold change and p_{adj} are given for the comparison between treatments with 100 mg L^{-1} naphthalene input after 2 hours (high-NAP 2h) and no-PAH controls after 24 hours.

NCBI RefSeq assembly GCF_000384415.1/ Locus tag	Putative function, *gene name(s)*	log2-fold change (Nap/cntrl.)	p_{adj}
**CYCPU_RS0111430**	(Hydroxy chromene-carboxylate) Isomerase, *phnD*[1,2]	-0.12	0.44
CYCPU_RS0111435	PAH dioxygenase component ferredoxin, *fer*, *phnA3*[1,2]	0.11	0.43
CYCPU_RS0111440	PAH dioxygenase component ferredoxin reductase, *pahA4*[1,2]	0.03	0.78
CYCPU_RS0111445	Hypothetical protein	0.27	0.01
CYCPU_RS0111450	Tryptophan synthase subunit β, *trpB*[1]	-0.12	0.36

CYCPU_RS0111455	Putative RHD β subunit, *orf7*[1]	1.97	1.74E-28
**CYCPU_RS0111460**	Ring cleavage dioxygenase, *rcd*, *phnC*[1,2]	-0.39	2.63E-3
CYCPU_RS0111465	RHD-3 β subunit, *rhd3β*, *phnA2*[1,2]	-0.52	1.72E-4
CYCPU_RS0111470	RHD-3 α subunit, *rhd3α*, *phnA1a*[1,2]	-0.47	2.30E-4
**CYCPU_RS0111475**	Aromatic dioxygenase large, *phnA1b*[1,2]	-0.04	0.82
**CYCPU_RS0111480**	Dihydrodiol dehydrogenas, *orf1*[1]	-0.33	1.29E-4
CYCPU_RS0111485	Hypothetical protein	-0.35	5.11E-3
**CYCPU_RS0111490**	RHD-PS1, Aromatic-ring-hydroxylating dioxygenase β, *rhdPS1β*	1.30	1.35E-13
**CYCPU_RS0111495**	RHD-PS1, Aromatic-ring-hydroxylating dioxygenase α, *rhdPS1α*	1.15	2.40E-22
CYCPU_RS0111500	DPR-2 regulator, *dpr2*	-0.06	0.56
CYCPU_RS0111505	RHD-9 α subunit, *rhd9α*	-0.15	0.45
CYCPU_RS0111510	RHD-9 β subunit, *rhd9β*	-0.65	1.16E-05
CYCPU_RS0111515	Short-chain dehydrogenase/reductase	-0.53	3.09E-5
CYCPU_RS0111520	Alcohol dehydrogenase	-0.50	3.26E-6
CYCPU_RS0111525	Outer membrane lipoprotein-sorting protein	-0.62	1.77E-9
**CYCPU_RS0111530**	Hypothetical protein	-1.26	8.85E-24
**CYCPU_RS0111535**	Multidrug efflux transporter, permease	-0.57	1.52E-6
**CYCPU_RS0111540**	Glycosyl hydrolase, BNR repeat	0.03	0.85
**CYCPU_RS0111545**	Alcohol dehydrogenase/quinone oxidoreductase	-0.14	0.40
**CYCPU_RS0111550**	Metabolite transporter superfamily, permease[2]	-0.10	0.63
**CYCPU_RS0111555**	RHD-2 α subunit, *rhd2α*[2]	-0.47	2.69E-3
**CYCPU_RS0111560**	RHD-2 β subunit, *rhd2β*[2]	-0.46	4.84E-09
**CYCPU_RS0111565**	Dioxygenase electron transfer component/ Carboxylate dehydrogenase	-0.65	5.87E-7
**CYCPU_RS0111570**	Glutathione S-transferase	-0.23	0.17
**CYCPU_RS0111575**	Hypothetical protein	0.39	0.02

**CYCPU_RS0111580**	Transglutaminase-like protein	0.05	0.72
**CYCPU_RS0111585**	Nitroreductase	-0.10	0.39
**CYCPU_RS0111590**	Outer membrane transport protein	0.54	7.29E-15

[1]Genes also part of "*phnA*-cluster" (Kasai *et al.*, 2003)
[2]Genes potentially regulated by DPR-2 regulator (Wang *et al.*, 2021)

References

Bagi A, Knapik K & Baussant T (2022) Abundance and diversity of n-alkane and PAH-degrading bacteria and their functional genes - potential for use in detection of marine oil pollution. *Sci Total Environ* **810**: 152238, DOI: 10.1016/j.scitotenv.2021.152238.

Dionisi HM, Lozada M, Marcos MS, Di Marzio WD & Loviso CL (2011) Aromatic hydrocarbon degradation genes from chronically polluted Subantarctic marine sediments. *Handbook of Molecular Microbial Ecology II: Metagenomics in Different Habitats,*(Bruijn FJd, ed.) p. 461-473. John Wiley & Sons Inc, New York.

Kanehisa M & Goto S (2000) KEGG: kyoto encyclopedia of genes and genomes. *Nucleic Acids Res* **28**: 27-30.

Kanehisa M, Sato Y & Morishima K (2016) BlastKOALA and GhostKOALA: KEGG tools for functional characterization of genome and metagenome sequences. *J Mol Biol* **428**: 726-731.

Kanehisa M, Sato Y, Kawashima M, Furumichi M & Tanabe M (2016) KEGG as a reference resource for gene and protein annotation. *Nucleic Acids Res* **44**: D457-D462.

Kasai Y, Shindo K, Harayama S & Misawa N (2003) Molecular characterization and substrate preference of a polycyclic aromatic hydrocarbon dioxygenase from *Cycloclasticus* sp. strain A5. *Appl Environ Microbiol* **69**: 6688-6697, DOI: 10.1128/aem.69.11.6688-6697.2003.

Liang C, Huang Y & Wang H (2019) *pahE*, a functional marker gene for polycyclic aromatic hydrocarbon-degrading bacteria. *Appl Environ Microbiol* **85**: DOI: 10.1128/AEM.02399-18.
Schoch CL, Ciufo S, Domrachev M, Hotton CL, Kannan S, Khovanskaya R, Leipe D, Mcveigh R, O'Neill K & Robbertse B (2020) NCBI Taxonomy: a comprehensive update on curation, resources and tools. *Database* **2020**.

Vogel AL, Thompson KJ, Straub D, App CB, Gutierrez T, Löffler FE & Kleindienst S (2023) Substrate-independent expression of key functional genes in *Cycloclasticus pugetii* strain PS-1 limits their use as markers for PAH biodegradation. *Frontiers Microbiol* **14**: DOI: 10.3389/fmicb.2023.1185619.
Wang W, Wang L & Shao Z (2018) Polycyclic aromatic hydrocarbon (PAH) degradation pathways of the obligate marine PAH degrader *Cycloclasticus* sp. strain P1. *Appl Environ Microbiol* **84**: e01261-01218, DOI: 10.1128/aem.01261-18.

Wang W, Shao Z & Zhang X-H (2021) An intracellular sensing and signal transduction system that regulates the metabolism of polycyclic aromatic hydrocarbons in bacteria. *mSystems* **6**: e00636-00621, DOI: 10.1128/mSystems.00636-21.

Wang Y, Lau P & Button DK (1996) A marine oligobacterium harboring genes known to be part of aromatic hydrocarbon degradation pathways of soil pseudomonads. *Appl Environ Microbiol* **62**: 2169-2173.
